# Unusual Influenza A Viruses in Bats

**DOI:** 10.3390/v6093438

**Published:** 2014-09-17

**Authors:** Andrew Mehle

**Affiliations:** Department of Medical Microbiology and Immunology, University of Wisconsin-Madison, Madison, WI 53706, USA; E-Mail: amehle@wisc.edu; Tel.: +1-608-263-1978

**Keywords:** influenza virus, bats, cross-species transmission, *Orthomyxoviridae*, host range

## Abstract

Influenza A viruses infect a remarkably diverse number of hosts. Two completely new influenza A virus subtypes were recently discovered in bats, dramatically expanding the host range of the virus. These bat viruses are extremely divergent from all other known strains and likely have unique replication cycles. Phylogenetic analysis indicates long-term, isolated evolution in bats. This is supported by a high seroprevalence in sampled bat populations. As bats represent ~20% of all classified mammals, these findings suggests the presence of a massive cryptic reservoir of poorly characterized influenza A viruses. Here, we review the exciting progress made on understanding these newly discovered viruses, and discuss their zoonotic potential.

## 1. Introduction

Bats are well-known reservoirs for viruses with zoonotic potential. Spillover of virus from bats to humans is thought to have caused acute infections by the paramyxoviruses Hendra and Nipah, severe acute respiratory syndrome (SARS)-coronavirus, Australian bat lyssavirus, and the filoviruses Marburg and Ravn [1,2,3,4,5,6,7,8,9]. Bats may have also been the ancestral source of hepatitis C, hepatitis B, mumps, and GB viruses that are endemic in humans [10,11,12]. Influenza A virus can now be added to this growing list; two new lineages of influenza A viruses were recently detected in the little yellow‑shouldered bat (*Sturnira lilium*, family Phyllostomidae), in Guatemala, and the flat-faced fruit‑eating bat (*Artibeus jamaicensis planirostris*, family Phyllostomidae), in Peru [13,14].

Influenza A virus is a remarkably promiscuous virus. Viruses circulating in birds, pigs and people garner the most attention, given their impact on public health, yet influenza virus naturally infects a diverse array of animals. These hosts include dogs, horses, cats, non-human primates, cattle, seals, whales, guinea pigs, ferrets, mink, giant pandas, pikas, raccoon dogs, anteaters, camels, and penguins (reviewed in [15,16,17]). The detection of influenza A virus in bats, which represent ~20% of all known mammals, dramatically expands the host range of this virus.

Influenza A virus is a member of the *Orthomyxoviridae* family with a segmented genome composed of eight single-stranded negative-sense RNAs [18]. The viral genome exploits splicing, frame-shifting and leaky scanning to code for at least 14 different proteins. Influenza viruses are classified into subtypes based on their two major surface proteins, hemagglutinin (HA) and neuraminidase (NA). Prior to the identification of influenza virus in bats, 16 HAs and nine NAs had been identified, circulating primarily in avian reservoirs. Of these, only H1N1, H2N2, and H3N2 are known to have caused pandemics in humans.

The viruses in bats, H17N10 in the little yellow-shouldered bat and H18N11 in the flat-faced fruit-eating bat, are completely new subtypes that are evolutionarily distinct from all other circulating strains [13,14,19]. Three H17N10 genomes (A/little yellow-shouldered bat/Guatemala/164/2009, A/little yellow-shouldered bat/Guatemala/153/2009, A/little yellow-shouldered bat/Guatemala/060/2010), and one H18N11 genome (A/flat-faced bat/Peru/033/2010) were reconstructed by RT-PCR. Each of the bat genomes maintains the same genomic architecture and coding potential of other influenza A viruses. H17N10 was initially detected in rectal swabs, and subsequently in liver, kidney, intestine, and lung tissue [13]. H18N11 RNA was also first detected in a rectal swab, and then exclusively in intestinal tissue. Infectious isolates were not recovered from these animals, but the abundance of genomic RNA in the intestine suggests that tissue tropism in bats is more akin to birds, in which influenza virus replicates in the intestinal tract, than to most mammals, in which infections occur primarily in the respiratory tract. Reports from 1979–1982 also claimed isolation of influenza A virus from bats (A/bat/Alma-Ata/73/1976), as well as the detection of antibodies against influenza virus in bats [20,21,22]. However, this earlier bat virus showed immuno-reactivity to sera raised against human H3N2 viruses, and the bat sera recognized human H2N2 and H3N2 viruses, indicating that if these were *bona fide* influenza virus infections in bats, they were distinct from the newly described viruses from South American bats.

H17N10 and H18N11 viruses were identified in bats of different species located over 3000 km apart and over multiple years. The two subtypes are more closely related to each other than to any other influenza A virus, yet they display a high degree of divergence [13,14]. This suggests that replication and diversification of these viruses occurred over a significant period of time. The seroprevalence was high in South American bat populations (up to 50%) [14], indicating that infections can be common. Although, surveillance in Central European bats failed to detect any influenza virus in over 1300 animals, suggesting that not all bat populations are infected [23]. Thus, bats might represent a vast migratory reservoir of novel influenza viruses. A major concern with the discovery of these viruses, and many other unique influenza strains, is their potential to cause disease in humans. Even though infectious isolates or recombinant versions of H17N10 and H18N11 have not yet been reported, significant progress has been made in understanding the biology and replicative capacity of these viruses.

## 2. Zoonotic Potential

Transmission of influenza viruses into a new host and subsequent adaptation for sustained replication occurs through a combination of mutations to individual viral genes and reassortment of entire gene segments. Reconstruction of the evolutionary history of an individual viral strain can be used to detect these events. Phylogenetic analysis indicates an ancient origin for H17N10 and H18N11 lineages [13,14]. NA and the “internal” gene segments from bat viruses (PB2, PB1, PA, NP, NS, M) form outgroups to the same genes from all other extant influenza A viruses. H17 and H18 are more closely integrated into existing HA phylogenies, forming a monophyletic group with H1, H2, H5 and H6. It was originally hypothesized that the closer relatedness of H17 and H18 to existing HAs, especially compared to the outgroup status of the remainder of the bat genes, was indicative of past reassortment of HA genes between bat and avian viruses [13]. However, a more recent analysis invokes a large-scale sweep of internal gene segments in avian viruses, drastically reducing genetic diversity for all internal genes and inflating the impact of the outlier position of the bat genes [19]. In this scenario, the position of H17 and H18 in relation to other viruses is consistent with long-term evolution solely in bats and need not be the result of reassortment. The evolutionary reconstructions suggest an isolated trajectory in bats for H17N10 and H18N11, implying little influx of genomic diversity from other influenza strains in different hosts. They further suggest that cross-species transmission of these viruses out of bats is rare, if it occurs at all, and has not yet resulted in sustained infections. Of course, the current analysis is limited, with only four viral genomes from two bat strains available for comparison, and earlier reports of influenza virus in bats involved strains much more like human influenza strains. Additional surveillance is needed to determine the distribution of influenza viruses in bats and whether any new bat isolates might exhibit signs of gene flow between species.

There are two main barriers to cross-species transmission and the establishment of influenza zoonoses [24]: entry, mediated by HA (discussed below), and genome replication and transcription, performed by the viral RNA-dependent RNA polymerase (RdRP). The viral RdRP is composed of the subunits PB1, PB2, and PA. The RdRP assembles large complexes with viral RNA and the viral nucleoprotein (NP) to transcribe and replicate the genome [25]. Species-specific RdRP activity is an impediment to host switching. RdRPs derived from avian influenza virus generally function poorly in mammalian cells [26,27], possibly due to a potent restriction factor present in human cells that selectively impairs avian-derived polymerases [28]. A single mutation in the PB2 subunit, conversion of the avian-signature glutamic acid residue at position 627 to the human-signature lysine residue (E627K), is sufficient to overcome restriction in human cells [26,28,29,30,31]. Other mechanisms that escape restriction include second-site suppressor mutations and alterations in the nuclear import sequence of the PB2 subunit, as well as reassortment involving the PA subunit [32,33,34,35,36,37]. The block to polymerase function is strong enough to severely impair viral replication and transmission in animal models [29,31,38,39].

Polymerase proteins and NP in bat viruses contain conserved active site residues and many other key features required for function. A structure of the N-terminus of H17N10 PA shows that this region contains an endonuclease domain, as is the case in other PA genes [40,41,42]. Surprisingly, the bat PB2 genes contain neither lysine nor glutamic acid at PB2 position 627, but an unprecedented serine–the only viruses known to do so. Despite this unusual polymorphism, bat polymerases are highly active in human and avian cells [13,14,43]. Introduction of the bat-like PB2 S627 into an avian influenza virus polymerase is sufficient to overcome restriction in human cells [44]. We have also shown that bat cells restrict the activity of avian polymerase, targeting PB2 E627 from an avian-origin polymerase in the same fashion as human cells [45]. The bat polymerase and NP support high-titer replication of chimeric viruses in cells from a number of mammals, including dogs, pigs, humans and bats [43]. The bat polymerase appears well adapted to function in multiple species of bats, suggesting that it would not necessarily preclude cross-species transmission.

In addition to viruses emerging out of bat populations, multiple groups have tested whether avian and human influenza virus could possibly move into bats. To date, 15 bat cell lines from nine different species were all shown to be susceptible to infection by human influenza viruses [45,46,47]. These included cells from New World and Old World bats, frugivorous and insectivorous animals, multiple tissue types, and adult and embryonic sources. In addition, many cells were also infectible by avian, swine, and mouse-adapted viruses [43,46]. Remarkably, influenza B virus also replicated in bat cells [45]. Bat cells can be co-infected by two different influenza strains to produce reassortant virus [47]. However, the reassortment potential of H17N10 and H18N11 may be limited given that several bat viral genes and genome packaging elements are incompatible with those from a non-bat virus [43]. Thus, while in a lab setting there does not appear to be an intrinsic block to the replication of diverse influenza viruses in bat cells, it is important to determine whether bats are ever infected by human viruses in nature, as suggested earlier [20,21,22]. If so, this would open the possibility for production of reassortant viruses with altered host range and pathogenicity compared to their parental counterpart.

## 3. When Is an HA Not an HA?

Influenza virus attachment and entry are performed by HA [48]. HA is studded on the surface of the virion as a homotrimer. HA contains a membrane-distal globular head supported by an extended stem that inserts into the viral membrane. HA is post-translationally cleaved in the stem region to yield HA1 and HA2 subunits. A discrete receptor binding site (RBS) in the globular head of HA1 recognizes receptors on the surface of target cells during attachment, whereas the stem in HA2 functions primarily during fusion and entry. A hallmark of influenza virus HA is the use of sialic acid moieties as receptors for attachment to the surface of target cells. Species-specific variations in sialic acid structures are a major barrier to cross-species transmission; α2,3-linked sialic acids dominate at the site of infection in avian hosts compared to α2,6-linked sialic acids that are present in mammalian hosts [48]. HA evolves to recognize these distinct sialic acid variants, impacting tissue tropism within a host and the capacity for transmission between hosts, or even across species [49,50,51]. It was therefore surprising when initial analysis of both H17 and H18 HA showed that these proteins failed to recognize classical sialic acid receptors, a large number of naturally occurring sialosides, or any of over 600 glycans [14,52,53]. Additionally, recombinant H17 was unable to bind to MDCK cells, a cell type with abundant sialylated glycans commonly used to propagate high titer influenza virus stocks [52]. This is not because sialic acids are absent or inaccessible to influenza virus on bat cells, as we have shown that infection of bat cells by a prototypical human-origin influenza virus requires sialic acids on the cell surface [45].

**Figure 1:**
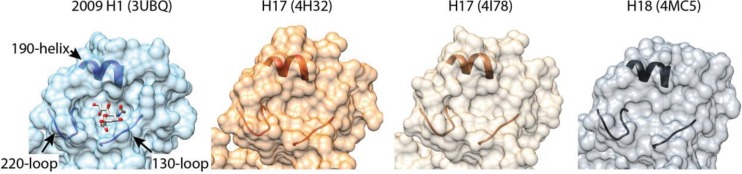
Altered receptor-binding sites in H17 and H18 HA. A binding cavity defined by the 130-loop, the 220-loop and the 190-helix is occupied by sialic acid in 2009 H1 HA [56]. Polymorphisms surrounding the binding cavity and the absence of conserved binding-site residues dramatically flatten the binding pocket in structures of H17 and H18. H17 and H18 fail to bind canonical sialic acid receptors (see text for details).

Structures of H17 and H18 help explain why these proteins do not bind sialic acid. The overall topology of H17 and H18 is remarkably similar to other HA trimers with an easily recognizable globular head and extended stem, despite only 45% sequence identity, on average, to other HA proteins [14,52,53]. H17 and H18 are most closely related to each other, yet, even there, the sequence identity is only 60%. This high degree of variability is consistent with the accelerated diversification of HA due to strong positive selection, including evasion of immune responses [54]. The structures of H17 and H18 differ most from canonical HA at the RBS. The RBS of canonical HA proteins is composed of a shallow binding cavity where sialic acid is flanked by key structural elements in the 130-loop, the 220-loop and the 190-helix (Figure 1) [55]. Conserved aromatic residues line the base of the cavity and critical amino acids in the loops and helix form the edge of the binding cavity. The amino acids on the edge of the cavity vary across HA subtypes to accommodate species-specific differences in sialic acid structure. Mutations in this region have also recently been shown to be important for aerosol transmission of H5-typed viruses in the ferret model [50,51].

Most of the key receptor-determining residues lining the edge of the RBS residues are not conserved in H17 or H18. Additional changes surrounding the RBS flatten the structure and almost eliminate the binding cavity, providing a molecular rationale for the altered receptor binding properties (Figure 1). Docking simulations predict that a model sialic acid will clash with the variant RBS in H17 [53]. Indeed, the presence of an aspartate at position 136 in the 130-loop of H17 and H18 would cause electrostatic repulsion of sialic acid in the binding cavity. The structures also reveal other changes in the HA trimer that might affect function and stability, notably the unusual exposed position of the fusion peptide at the base of the H17 stem [52]. Several questions immediately arise: What receptor or receptors are used by H17 and H18? Is the receptor a unique sialoside or glycan, another cell-surface moiety, or have H17 and H18 evolved to use protein receptors as has been suggested [52]? How does this impact the host range of bat virus and their capacity for reassortment with extant influenza viruses (and the inability so far to grow these viruses in the lab)? Do H17 and H18 mediate fusion in a similar pH-dependent fashion as other HA trimers? Conflicting reports on the pH-sensitive stability of H17 raise the possibility of alternative routes of entry and fusion triggers [52,53]. In addition, if H17 and H18 do not function as prototypical hemagglutinins, do bat N10 and N11 function as neuraminidases? Recent biochemical and structural studies of N10 and N11 have largely answered this last question.

## 4. An NA by Name Alone

Whereas HA mediates attachment and entry, NA functions primarily during viral assembly and release. NA cleaves sialic acids present on the surface of cells or virions to prevent premature binding by HA to the originally infected cell and aggregation with other virions, ensuring efficient exit and dispersal. Like HA proteins from bat influenza virus that do not function as classic hemagglutinins, the bat NA proteins do not appear to behave as classical neuraminidases. Neither N10 nor N11 displayed sialidase activity against a model substrate or any of a large number of glycans [14,57,58].

**Figure 2:**
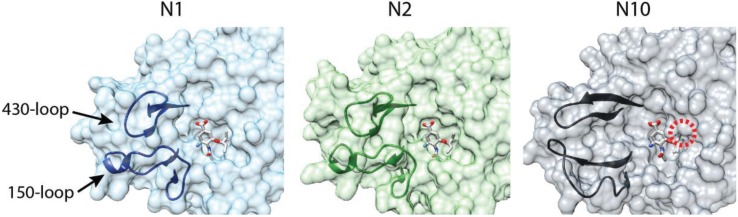
Comparison of the NA active site. Surface representations of the active sites for N1 (2HU4) and N2 (2QWK) in the presence of the neuraminidase inhibitor oseltamivir [59,60]. The substrate binding pocket is framed by the 150- and 430-loops. The bat influenza virus N10 active site (4FVK) is shown with oseltamivir from the N1 and N2 structures superimposed. The substrate binding pocket is dramatically opened relative to N1 and N2. A predicted clash between N10 and the modeled oseltamivir is denoted with a red circle.

Multiple crystal structures for N10 and N11 have begun to explain this difference in substrate specificity [14,57,58]. NA from previously characterized subtypes assembles as a tetramer with a “box-shaped” ectodomain supported by transmembrane domains that insert into the viral lipid envelope. The enzymatic activity is localized to the ectodomain. N10 and N11 assume this same global architecture. The primary difference lies within the active site. Few of the residues involved in substrate binding and catalysis for all other NAs are conserved in N10 and N11. Additionally, few of the so-called second-shell residues that stabilize the active site are conserved. The 150- and 430-loops that frame the active site are shifted away, substantially enlarging the size of the substrate binding pocket (Figure 2). Unexpectedly, the 150-loop in N10 participates in an unusual inter-chain interaction not present in other NAs.

Superimposing sialic acid from prior NA structures onto N10 or N11 shows that even in these larger binding pockets the classical sialic acid substrate would clash with residues lining the pocket [14,58]. The same is true for the neuraminidase inhibitor oseltamivir; overlaying oseltamivir from N1- or N2-liganded structures onto N10 predicts a clash in the active site (Figure 2). A similar topology and predicted clash is present in structures of N11, although less pronounced than that of N10 [14]. The enlarged binding pocket, the re-positioning of the 150-loop, which is important for inhibitor binding [60], and the predicted clash make it unlikely that current neuraminidase inhibitors will function against the bat NAs. The substrate(s) for N10 and N11 remains unknown. Analogous to the opposing receptor-binding and receptor-destroying activities of HA and NA in all other influenza viruses, the substrate may be the same unidentified molecule(s) that is used by H17 and H18 as a receptor. Alternatively, if the bat influenza viruses have evolved to utilize a completely new class of receptor, the receptor-destroying activity of NA may be superfluous and N10 and N11 might fulfill a different role during replication.

## 5. Perspective

There are over 1200 bat species, of which only a vanishingly small number have been surveyed for influenza virus infection. It seems highly likely that more viral isolates will be identified, and possibly additional influenza A subtypes. It also remains possible that focused efforts will identify viruses that more closely resemble those from avian or other mammalian reservoirs. Detection of naturally occurring infections in bats will begin to address the impact of these infections on bat health, the sites of replication, the mode of transmission between animals, and whether these viruses pose a significant threat to human health. Isolation of an infectious clone, or one produced by reverse genetics, will start to unravel the unusual biology and replication strategy that these viruses appear to employ. Especially important is the identification of the receptor used by H17 and H18 viruses. These findings will have implications for how influenza viruses in general adapt to and replicate in novel hosts, and how bat viruses in particular impact the genetic diversity of strains circulating in avian and mammalian reservoirs.
